# Supplementary material for: Immuno-detection by sequencing enables large-scale high-dimensional phenotyping in cells
Source: Nat Commun. 2018 Jun 19;9:2384. doi: 10.1038/s41467-018-04761-0 (PMC6008431; doi:10.1038/s41467-018-04761-0)
Supplement: Supplementary file 1 — Supplementary Information [file 41467_2018_4761_MOESM1_ESM.pdf]

Supplementary information

Immuno-Detection by sequencing (ID-seq) enables large-scale high-dimensional phenotyping in cells.

Jessie A.G. van Buggenum[1], Jan P. Gerlach[1], Sabine E.J. Tanis[1], Mark Hogeweg[1,2], Pascal W.T.C. Jansen[3], Jesse Middelwijk[4], Ruud van der Steen[4], Michiel Vermeulen[3], Hendrik G. Stunnenberg[3], Cornelis A. Albers[1,2] and Klaas W. Mulder[1,*]

Affiliations:

Department of Molecular Developmental Biology, Radboud Institute for Molecular Life Sciences, Radboud University, PO Box 9101, 6500 HB, Nijmegen, The Netherlands[1]. Department of Human Genetics, Donders Institute for Brain, Cognition and Behaviour, Radboud University Medical Center, PO Box 9101, 6500 HB, Nijmegen, The Netherlands[2]. Department of Molecular Biology, Radboud Institute for Molecular Life Sciences, Radboud University, PO Box 9101, 6500 HB, Nijmegen, The Netherlands[3]Biolegio BV, PO Box 91, 6500 AB Nijmegen, the Netherlands[4].

* To whom correspondence should be addressed (k.mulder@science.ru.nl)

Supplementary Notes

Supplementary Note 1. DNA-tag and primer design

DNA-tagging of antibodies can serve as a multiplexed and high-throughput readout for epitope detection. By using the PCR-based amplification of DNA, we produced multi- barcoded sequencing samples that allow counting of each unique antibody-DNA tag and annotating hundreds of samples. Supplementary Data 3 contains all tag and primer sequences. Here we describe our reasoning behind the DNA-tag and barcoded sequencing library design.

DNA-tag design.

We considered several practical requirements for our DNA-tag design. First, different DNA- tags need to be produced and coupled to different antibodies. The produced DNA needs purification before coupling to antibodies, using standard PCR purification columns that can purify DNA with lengths down to ~100 bp. To this end, we included barcodes that were on average 108 bp long. Second, each DNA-tag needs a unique “barcode” sequence, easily distinguishable after sequencing. Therefore, we designed a 10 bp long barcode-sequence (Barcode 1) with a minimum difference of 2 bp among the different barcodes. Third, we aimed for counting barcodes including corrections for PCR-amplification biases. For this, we included a 15 bp long unique molecular identifier (UMI) sequence. Finally, we should be able to sequence many ID-seq samples on an Illumina sequencer. We encountered a limitation of the Nextseq500 sequencer: when all reads have at a specific read-position the same base called, the sequencing run will stop because the sequencing chemistry in combination with the software used for base calling will throw a critical error. Therefore, we included a 'spacer' sequence of length 0 to 8 bp at the start of the sequenced region of the DNA-tag to introduce sample complexity. Considering these four requirements, the DNA-tags contain five specific sequences (Supplementary Figure 1a, Supplementary Data 3):

- Illumina adaptor and FR read 1 sequence; to get an extended sequence > 100bp ánd to include adaptor and primer needed for sequencing.
- A 'spacer' sequence of length 0 to 8 bp; to increase sample complexity.
- A unique molecular identifier (UMI) sequence of 15 bp; for unique barcode counting.
- A barcode sequence of 10 bp; antibody specific and with minimum two bp difference.
- Common reverse primer sequence of 19 bp; for PCR-based sample barcoding and for finding barcode and UMI sequences in reads (“anchor” sequence).

Sample-preparation primer design.

The released DNA-tags are prepared for sequencing via a two-step PCR protocol (see Materials and Methods). The first so-called ‘overhang PCR’ adds a DNA sequence with a sample-specific barcode to all released material ("Barcode 2", Supplementary Figure 1b,c). After pooling the barcoded DNA-tags, the second PCR reaction adds the Illumina "index" and the second adaptor sequences to the barcoded DNA (Supplementary Figure 1d).

Sequencing library design.

The final sequencing library contains ‘Illumina forward adapter,' spacer (0-8 bp), UMI, Barcode 1 (antibody-specific), common “anchor” sequence, Barcode 2 (sample-specific), Illumina reverse adaptor with sample index. (Supplementary Figure 1e, Supplementary Data 3).

Supplementary Note 2. Data Processing pipeline

To facilitate the analysis of ID-seq sequencing data, we developed a dedicated computational pipeline. Supplementary Figure 2 summarises the pipeline, showing input and output files, and six analysis steps. First, the bcl2fastq demultiplexing tool (v2.16.0.10) generates FASTQ files from bcl files. Second, our ID-seq R-package maps all reads from the FASTQ files to the 'anchor' sequence (Supplementary Figure 2e) to obtain the antibody barcode (Barcode 1), sample barcode (Barcode 2) and UMI sequences. Third, our R-package package allows duplicate read removal and barcode counting. Fourth, we match the barcode sequences to the antibody and sample respectively. Then, we use DESeq2 R-package to calculate normalisation factors. Finally, we developed a linear mixed effect model to determine the effect of a condition. More details on each of the analysis steps we discuss in the following paragraphs.

(I) Illumina demultiplexing and quality control.

The standard pipeline from Illumina allowed us to demultiplex the sequencing data, based on the index barcode that was added during PCR II of the sample-preparation (Supplementary Figure 2d). After demultiplexing, the quality of the sequences is evaluated using FASTQC tool[1]. The output from this tool will contain a “red flag,” which is common to all ID-seq experiments. Namely, all samples will include reads that contain a small part of the Illumina adaptor due to the inserts short length and spacer sequence (Supplementary Figure 2e).

(II) ID-seq demultiplexing using R-package ID-seq.

We developed an R-package that includes a function to “split” reads into UMI, Barcode_1 and Barcode_2 sequences. We reasoned that we could use the common “anchor” sequence (Supplementary Figure 2c,e) because it is present in all reads. Therefore, we use approximate matching of the reads to the known anchor sequence to split the reads. The function output is a tab separated file with UMI and barcode sequences, a log file with run information and a “.log” file with experiment information (number of reads and number of split reads per processed FASTQ file). We obtained a high percentage of ‘mapped’ reads to our ID-seq specific anchor sequence while allowing 10% mismatches (99 ± 0.4%).

(III) UMI analysis and barcode counts.

The 15 nucleotide long unique molecular identifier (UMI) is used to count tags per antibody in each sample. We observe very high percentage of unique reads in ID-seq experiments performed in our lab (94.7 ± 3.8 %, n=16 experiments). After removing duplicates, we count unique tags per antibody and sample. This UMI-based counting approach allows us to correct PCR amplification biases and count the released tags per antibody in each sample.

(IV) Barcode matching.

After obtaining tag counts per barcode, we continued with matching each barcode to a specific antibody and sample. We chose to allow no mismatches by matching known barcode sequences to the count table using R-package dplyr (function “left_join”).

(V & VI) Normalisation and analysis.

The count characteristic of ID-seq data allowed us to normalise counts with DESeq2 R- package[2] and to determine effects by generating mixed linear models that take into account the negative binomial distribution (Supplementary note 3).

Supplementary Note 3. Linear Mixed Effect Model description

We used a linear mixed effect model to test which treatments affected the protein level as measured with a specific DNA-tagged antibody. We used a negative binomial distribution to model the UMI counts Y_{acr} for a specific antibody a in replicate r of condition c. Here a conditon c can represent the control condition or a drug treatment. We use the tuple (a, c, r) to define an experiment. The model is used to test whether the antibody UMI counts for a specific treatment of interest t and antibody a is significantly different from the control condition, taking into account replicate experiments for the same treatment. We always apply the model to each antibody independently. However, we analyze all experiments for a given antibody jointly to improve the estimation of the negative binomial overdispersion parameter, which may be different for each antibody due to differences in efficiency. The effect of the treatment of interest t compared to control is modeled using a fixed effect $b_{c=t}$; the effect of all other treatments (relative to the control condition) is modeled using an overall treatment mean $\mu_{c\neq t}$ and a random effect $\beta_{c\neq t} \sim \mathcal{N}(0, \sigma_{c\neq t})$ for each treatment (other than the treatment of interest). As the sequencing depth varied for each experiment, we normalized using a library size factor L_{aer} [3,2].

We then have

$$Y_{acr} \sim \mathcal{NB}(\mu_{acr}, \sigma(\theta_a)),$$

where θ_a is the overdispersion parameter.

$$\begin{cases} \log\mu_{acr} = \mu_{\text{control}} + L_{acr} + \gamma_{\text{plate}(acr)} & c \text{ is control experiment} \\ \log\mu_{acr} = \mu_{\text{control}} + L_{acr} + \gamma_{\text{plate}(acr)} + b_{c=t} & c \text{ is treatment of interest } t \\ \log\mu_{acr} = \mu_{\text{control}} + L_{acr} + \gamma_{\text{plate}(acr)} + \mu_{c\neq t} + \beta_{c\neq t} & c \text{ is not control and not treatment of interest } t \end{cases}$$

Here μ_{control} is the mean of the control experiments; $\gamma_{\text{plate}(acr)}$ is a fixed effect (plate(acr) is the plate index of an experiment) that accounts for systematic plate effects. It can be seen that $\mu_{c\neq t}$ represents a mean treatment effect based on all treatments except the treatment of interest t. We are not explicitly interested in the value of $\mu_{c\neq t}$ or $\sigma_{c\neq t}$, but we consider these nuisance variables that aid in the estimation of the overdispersion parameter θ_a.

We used the lme4 packagev[4] in R to implement the model and estimate the parameters and statistical significance of $b_{c=t}$. Since we would like to know for each treatment if there is a significant effect compared to control, the model needs to be estimated separately for all treatments and all antibodies (each time a different treatment being the treatment of interest). Thus, in fact the parameter θ_a depends on the treatment of interest t and may vary slightly between different treatments of interest.

Supplementary Figures

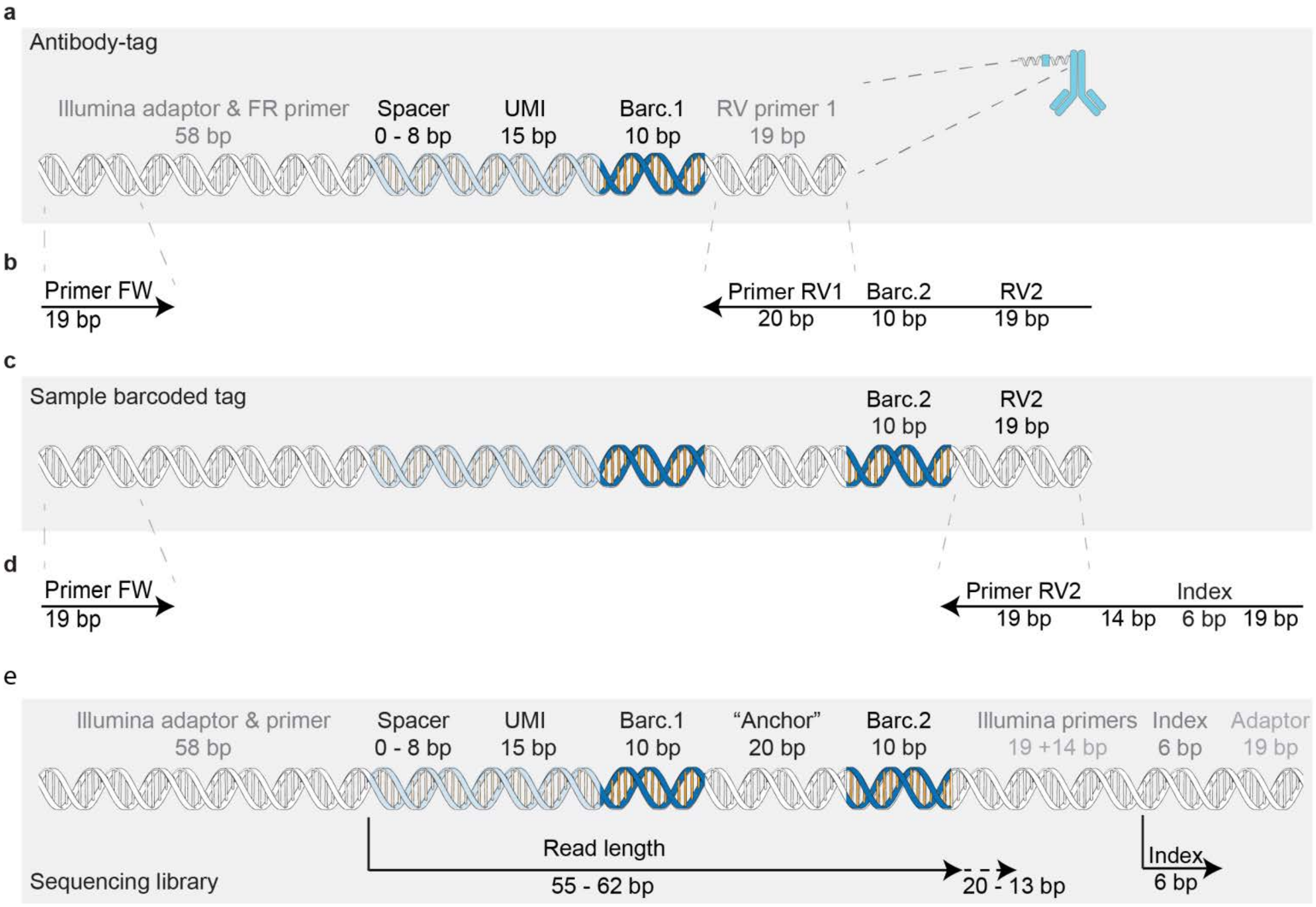

Supplementary Figure 1. Schematic overview of the DNA-tag and primer sequences. **(a)** The DNA-tag (antibody-tag) sequence includes a spacer (0 to 8 bp), a UMI and antibody-specific Barcode 1 (Barc.1) sequence. **(b)** After release of the DNA tags, a 19 bp forward primer and a 49 bp reverse primer is used to amplify the tag and include a sample-specific Barcode 2 (Barc.2) sequence. **(c)** The product of this PCR step includes the sequences from the DNA tag plus the sample-specific barcode and another reverse primer sequence. This sequence is the start of the Illumina adapter. **(d)** After pooling all bar-coded samples. A final PCR is performed using the forward primer and a custom-made unique reverse primer that is the actual illumine adapter and index sequence. **(e)** The product of the final PCR step of the sample preparation has a small insert between the adapters, which includes the spacer sequence, the UMI sequence, the Barcode 1 sequence, anchor sequence, and finally the Barcode 2 sequence. The index from the adapter is used as a barcode for the sequencing sample. Each sequencing run will result in reads that contain full UMI, Barcode 1, anchor and Barcode 2 sequences.

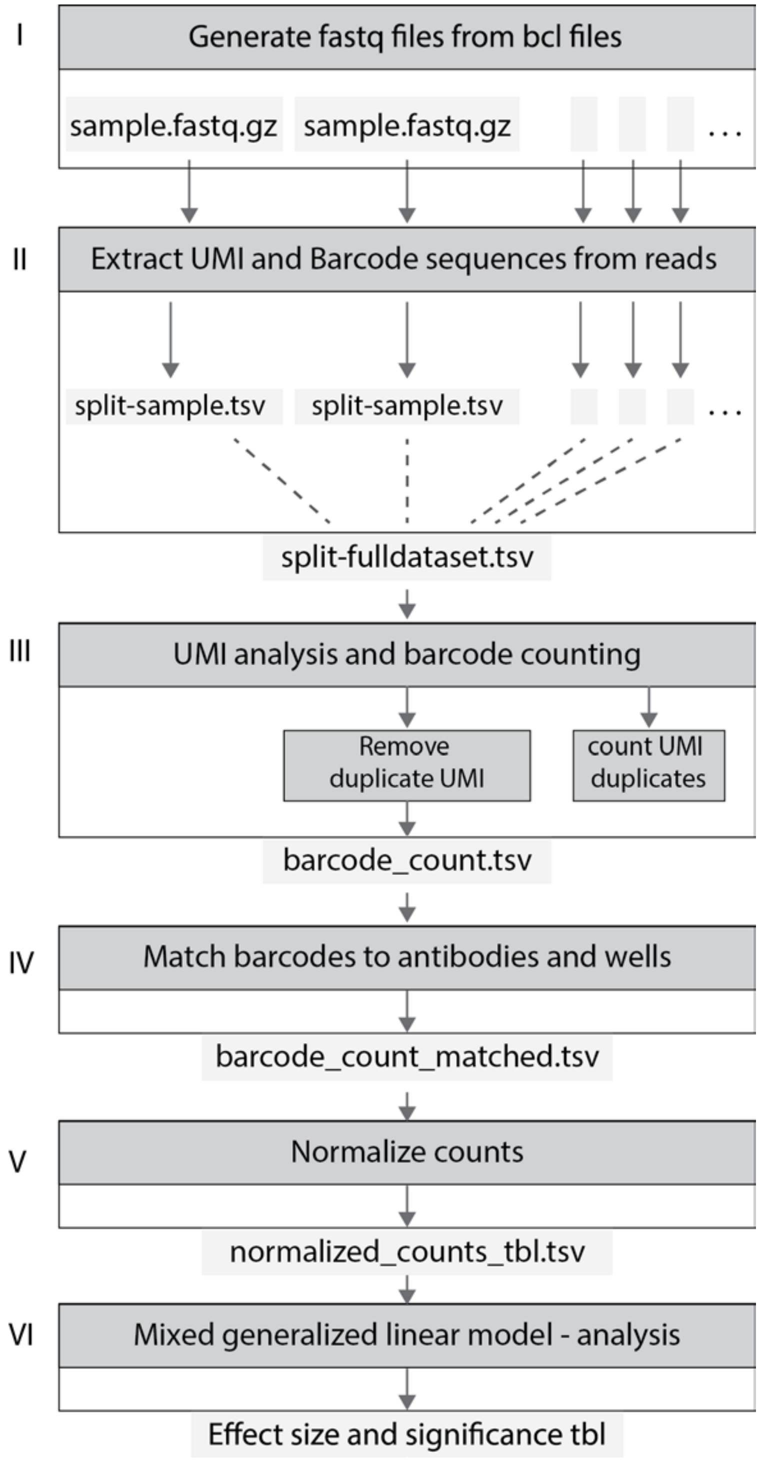

Supplementary Figure 2. Schematic overview of the analysis pipeline. To process and analyse ID-seq datasets, we set up an analysis pipeline consisting of six steps. First, bcl2fastq converts raw data to indexed FASTQ files (I). To extract barcodes from the reads, we created an R-package that includes a function split reads from FASTQ files (II). The split reads are used to determine duplicate rates and to count tags (III). The ID-seq package includes a function that matches barcodes to antibody and sample information based on two index files. Optionally, one can use the dplyr package to annotate the table manually (IV). We use the DESeq2 package to calculate normalisation factors for each sequencing library (V). The counts and the normalisation factors are utilized in a linear mixed effect model to determine the effectsize (so-called "Estimate") of a specific treatment (VI) and calculate significance of the effect using a likelihood ratio test (Supplementary Note 3).

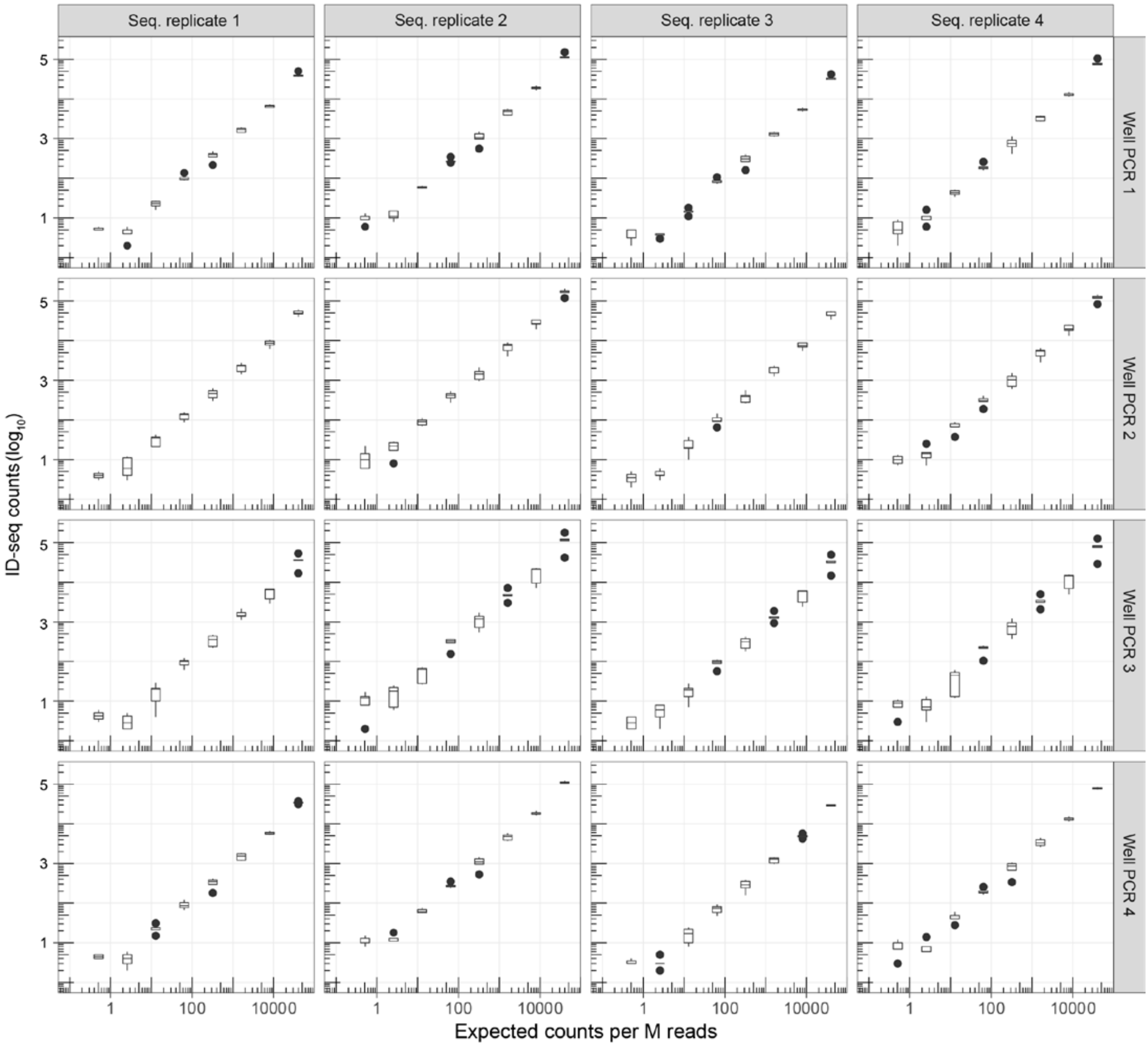

Supplementary Figure 3. UMI counts from spike-in DNA-tags shows dynamic range of count-based barcode measurement. A mix of spike-in template DNA tags was prepared using eight concentrations (n = 5 tags per concentration). Four 'well-PCR' reactions were performed, each adding a sample-specific DNA sequence to the DNA tags. Then, the four reactions were pooled for purification. Finally, this sample was prepared for sequencing via a second PCR, in quadruplicate (sequencing replicate 1 to 4). After sequencing, unique UMI sequences were counted. Each boxplot shows the counts from the five DNA-tags at that specific concentration. Over four orders of magnitude, the counts correlate with the expected input counts, for all four replicate sequencing samples. This illustrates how the ID-seq readout can have a dynamic range of four order of magnitude. (Boxplots with center line indicating median, bounds of boxes showing upper and lower quartile, and whiskers illustrating 1.5*inter-quartile range, n= 5)

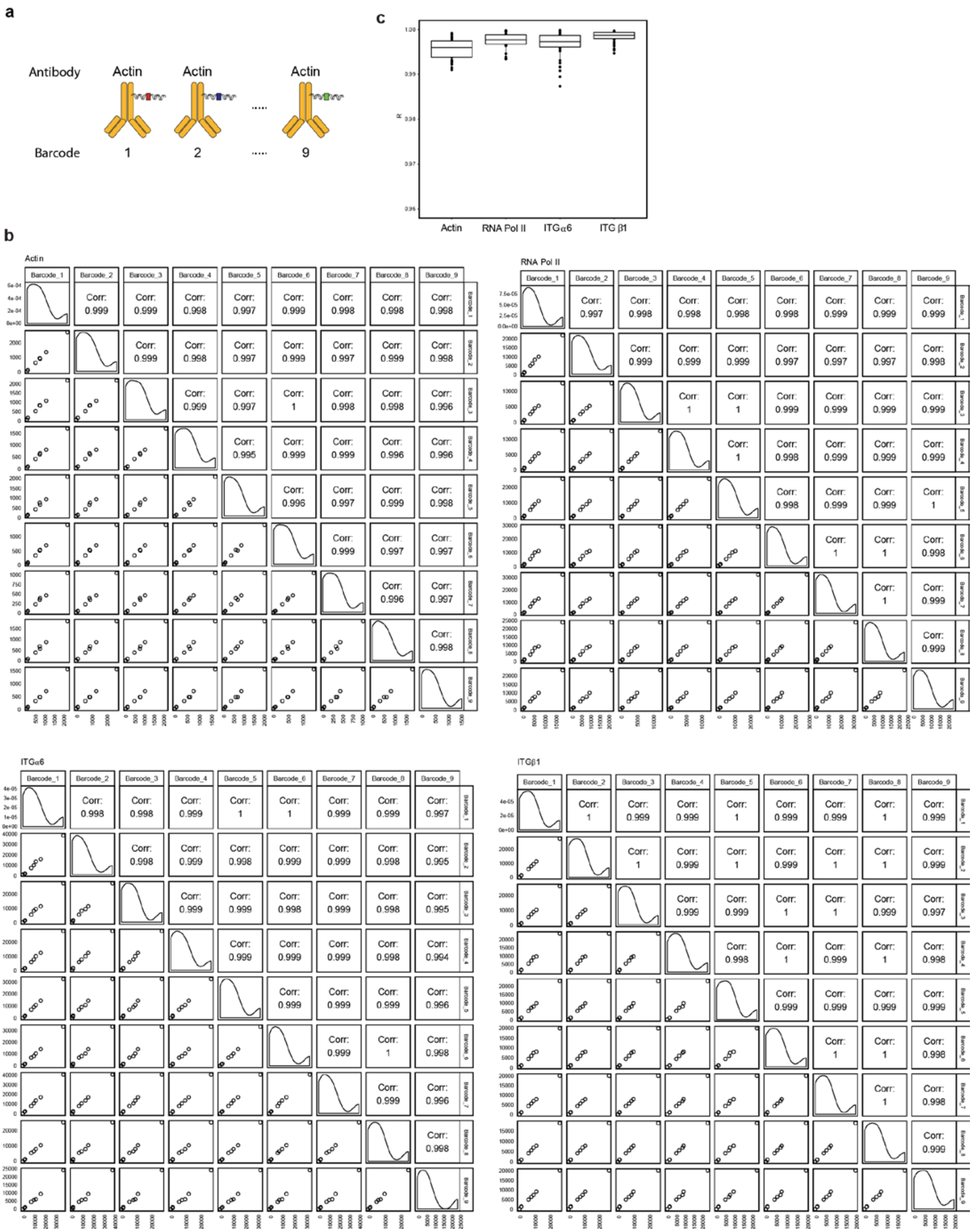

Supplementary Figure 4. Precision of ID-seq measurement determined by multi-labelling of antibodies. **(a)** Schematic overview of the principle to couple 9 barcodes to 1 antibody. **(b)** Correlation graphs of 9 barcode 'tags' from each antibody. Each barcode is an internal technical replicate of the immunostaining. **(c)** Boxplot of all correlations between the 9 barcodes per antibody. (Boxplots with center line indicating median, bounds of boxes showing upper and lower quartile, and whiskers illustrating 1.5*inter-quartile range)

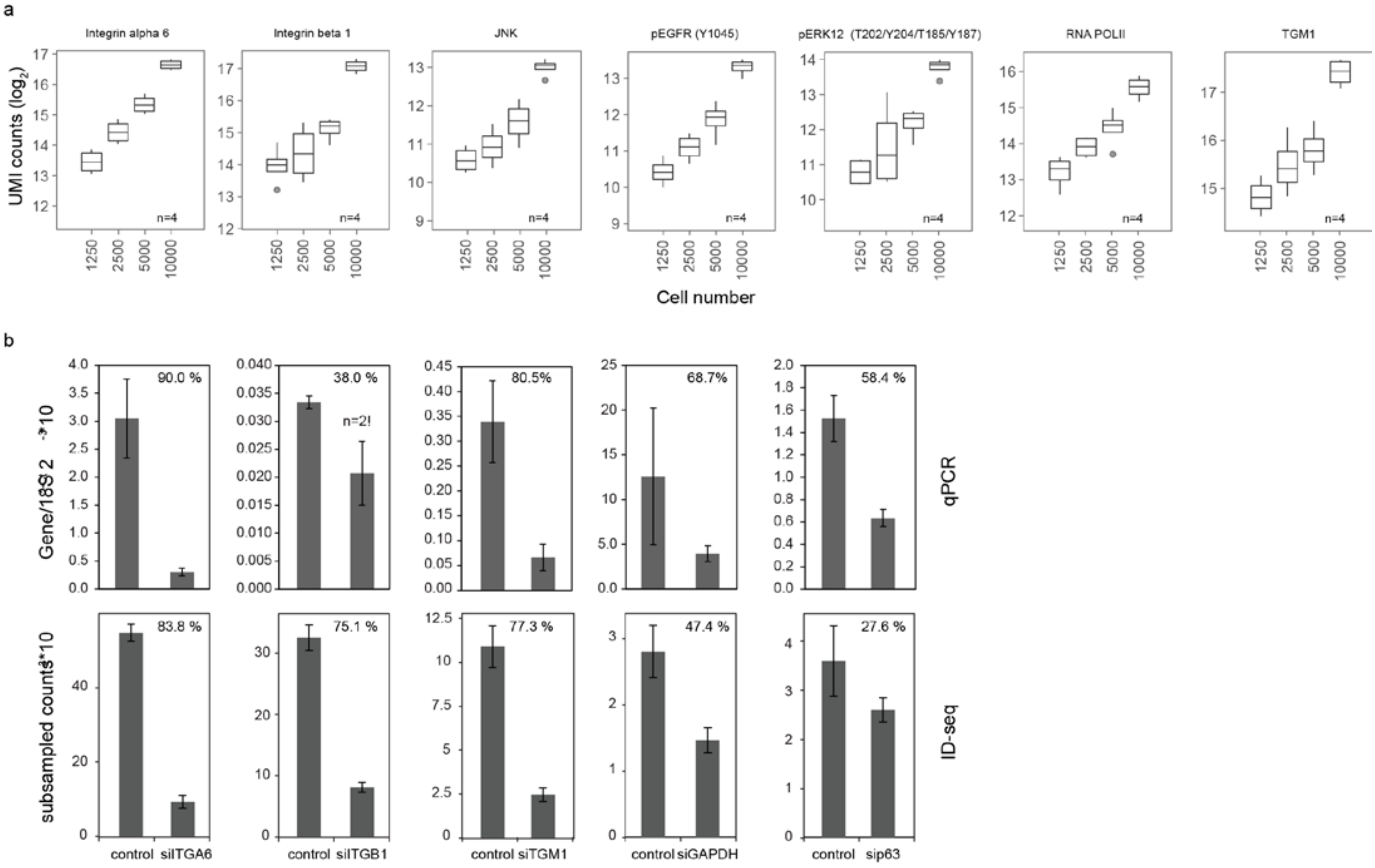

Supplementary Figure 5. ID-seq signal from different cell-numbers shows sensitive protein detection via DNA-tagged antibodies. (a) Keratinocytes were seeded in 96 wells plate at indicated numbers and grown for 24 hours. ID-seq signal from antibodies targeting indicated (phospho-)proteins (panel captions) shows log linear relation between seeded cell number and signal illustrating sensitive barcode detection of low cell numbers. (Boxplots with center line indicating median, bounds of boxes showing upper and lower quartile, and whiskers illustrating 1.5*inter-quartile range, n=4) (b) qPCR (see Supplementary Table 1 for primer sequences) and ID-seq show decreased signal after siRNA mediated knockdown of indicated proteins (mean and s.d., n= 3). Percentages show percentage of signal decrease.

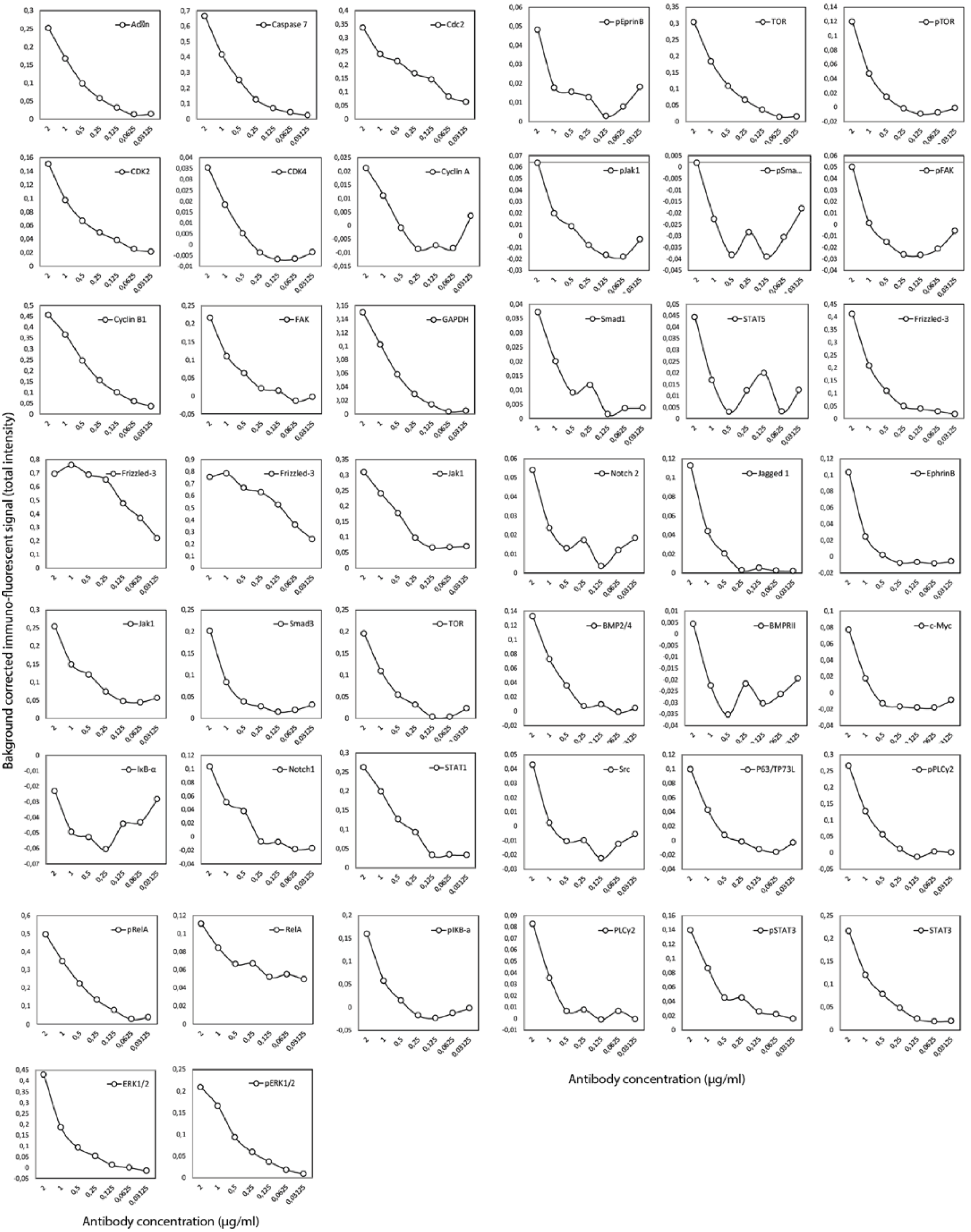

Supplementary Figure 6. Immuno-fluorescence signal of antibody dilution series of antibodies against indicated targets. Supplementary Data 1 gives an overview of all antibodies and their corresponding validation experiments.

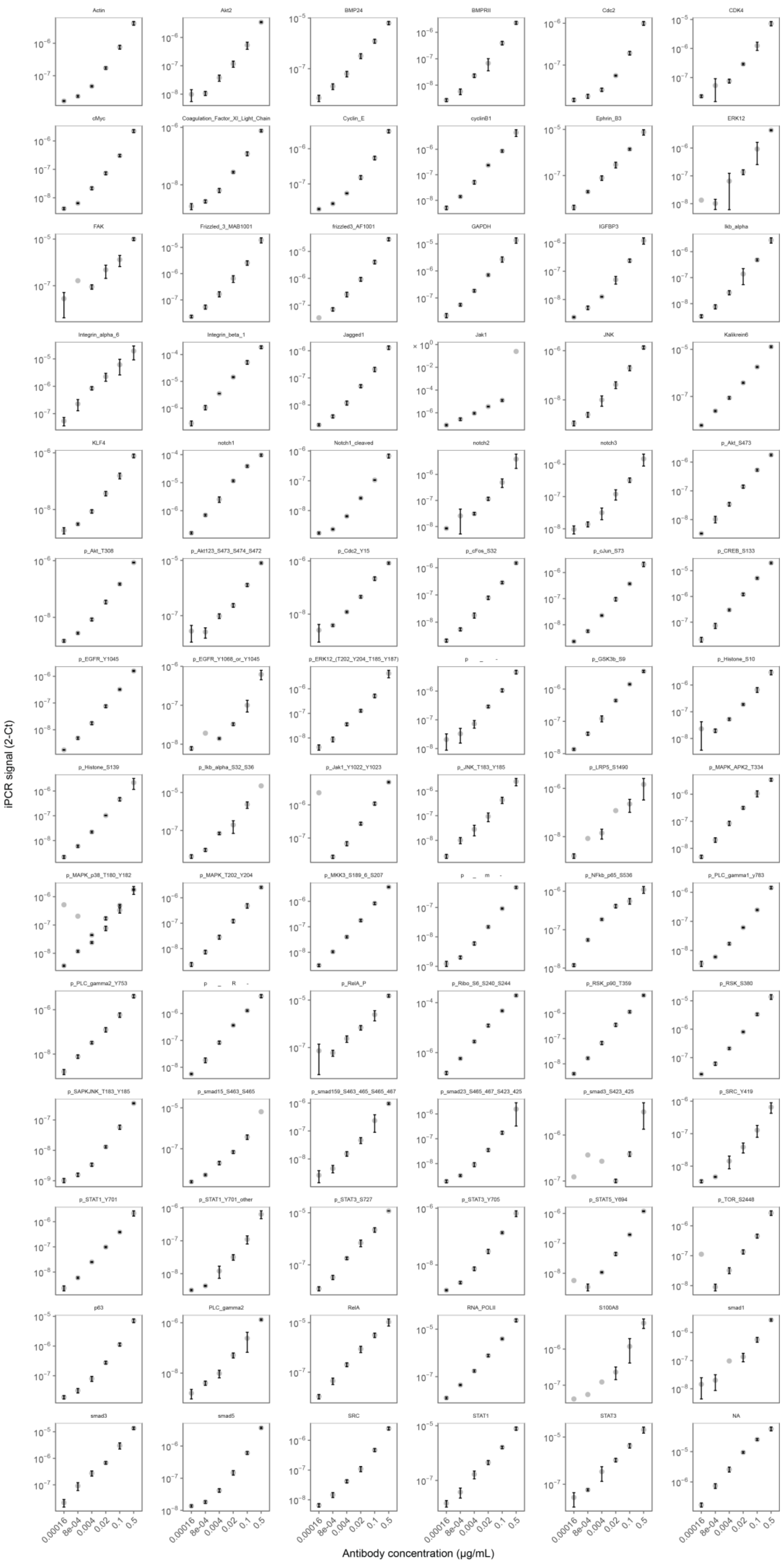

Supplementary Figure 7. Immuno-PCR signal of antibody-dilutions series of antibodies (panel captions) coupled to DNA-tags as described by van Buggenum et al. (Scientific reports, 2016). Supplementary Data 1 gives an overview of all antibodies and their corresponding validation experiments.

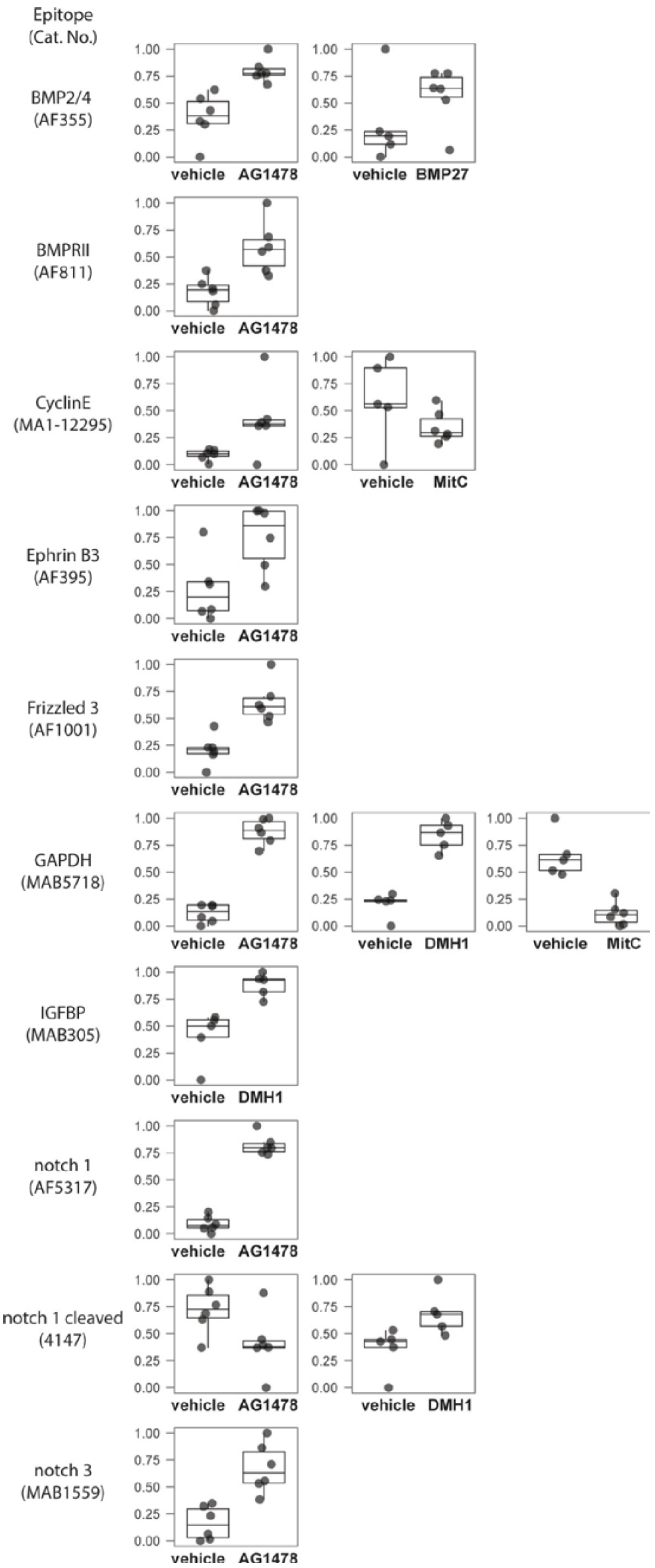

Supplementary Figure 8. Characterization of antibodies in ID-seq shows dynamic changes in skin stem cells. Boxplots with center line indicating median, bounds of boxes showing upper and lower quartile, and whiskers illustrating 1.5*inter-quartile range (n = 6). Skin stem cells were treated indicated compounds: AG1478 inhibits the EGFR. DMH1 inhibits BMP signaling. MitC and HydroxyU cause DNA damage and have antiproliferative capacities. Recombinant EGF and BMP2/7 activate EGFR and BMPR signaling, respectively. Plots show the scaled to minimum and maximum normalized counts of indicated antibodies (shown are target epitope and cat.no, see Supplementary Data 1 for further details).

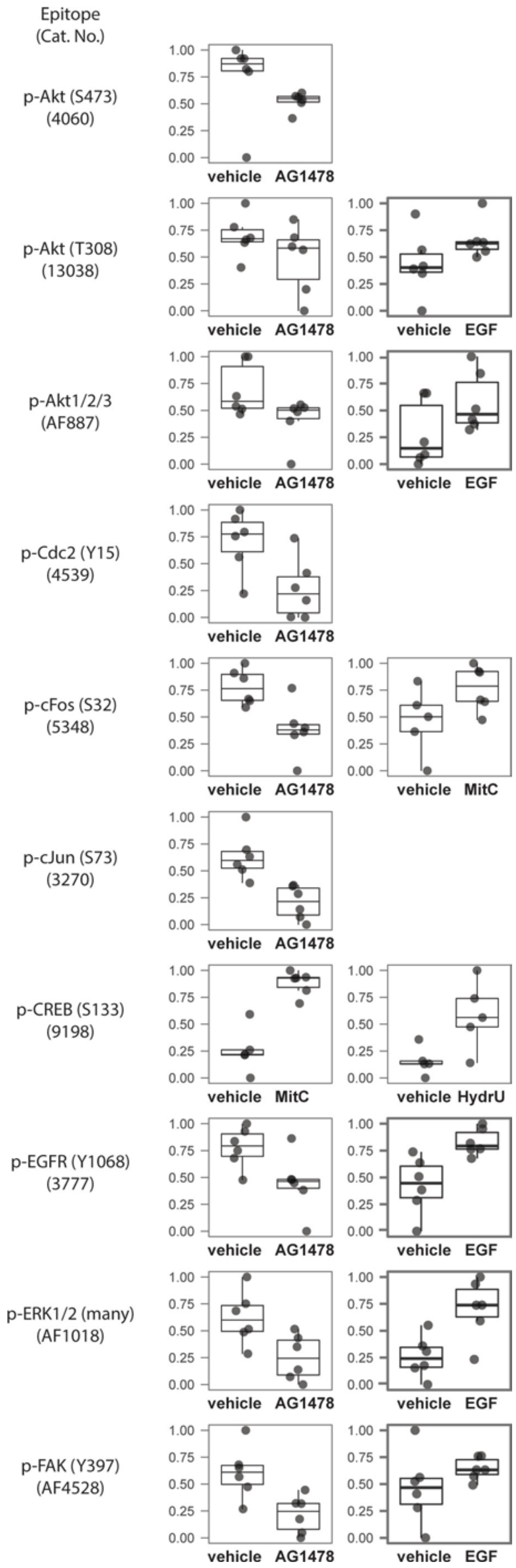

Supplementary Figure 8 continued

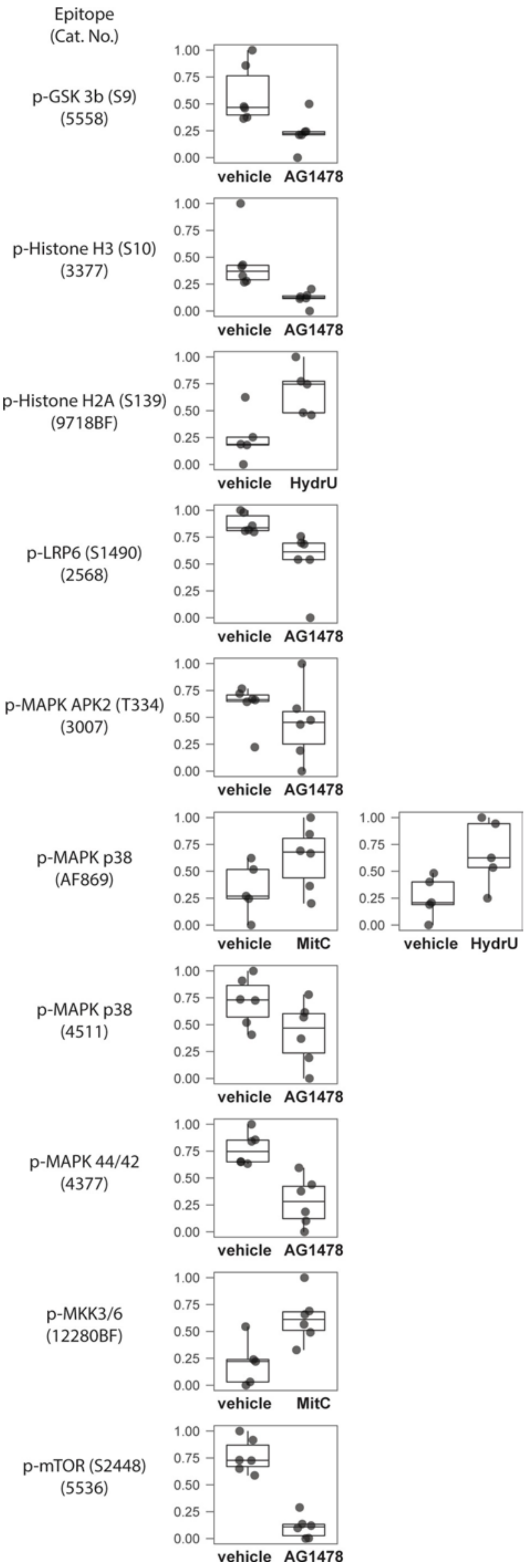

Supplementary Figure 8 continued.

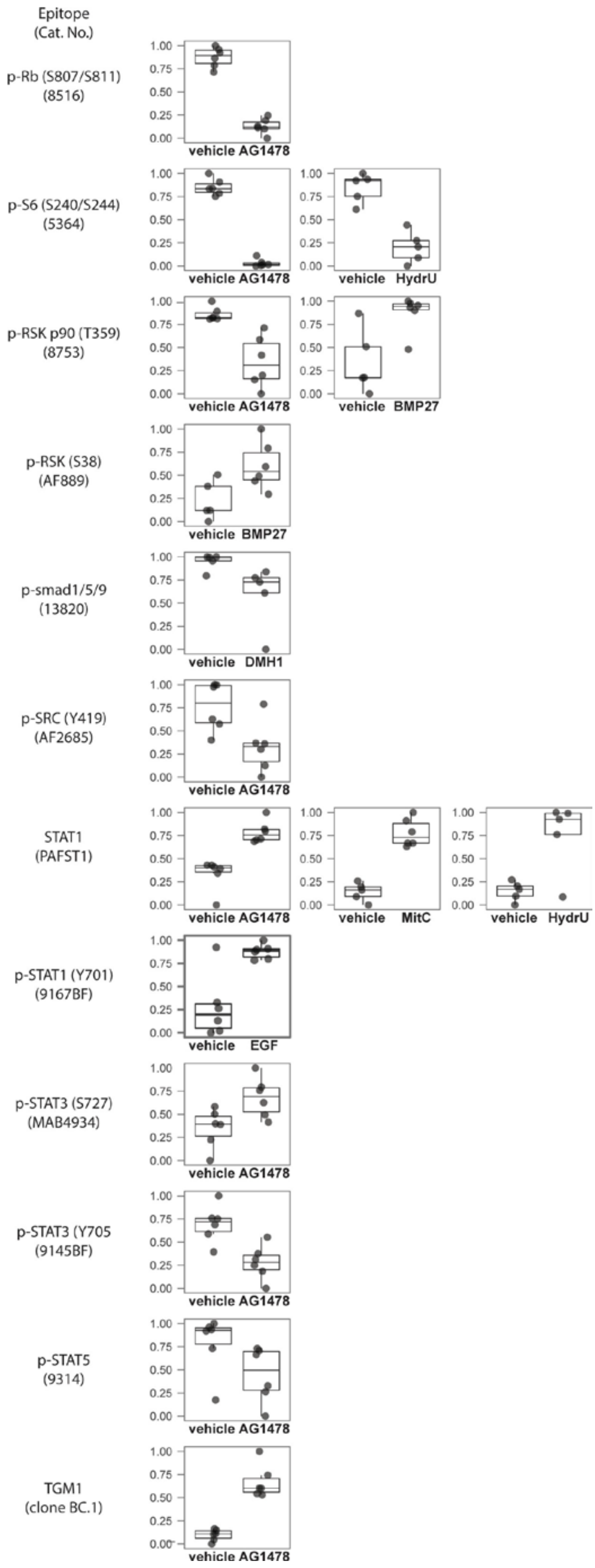

Supplementary Figure 8 continued

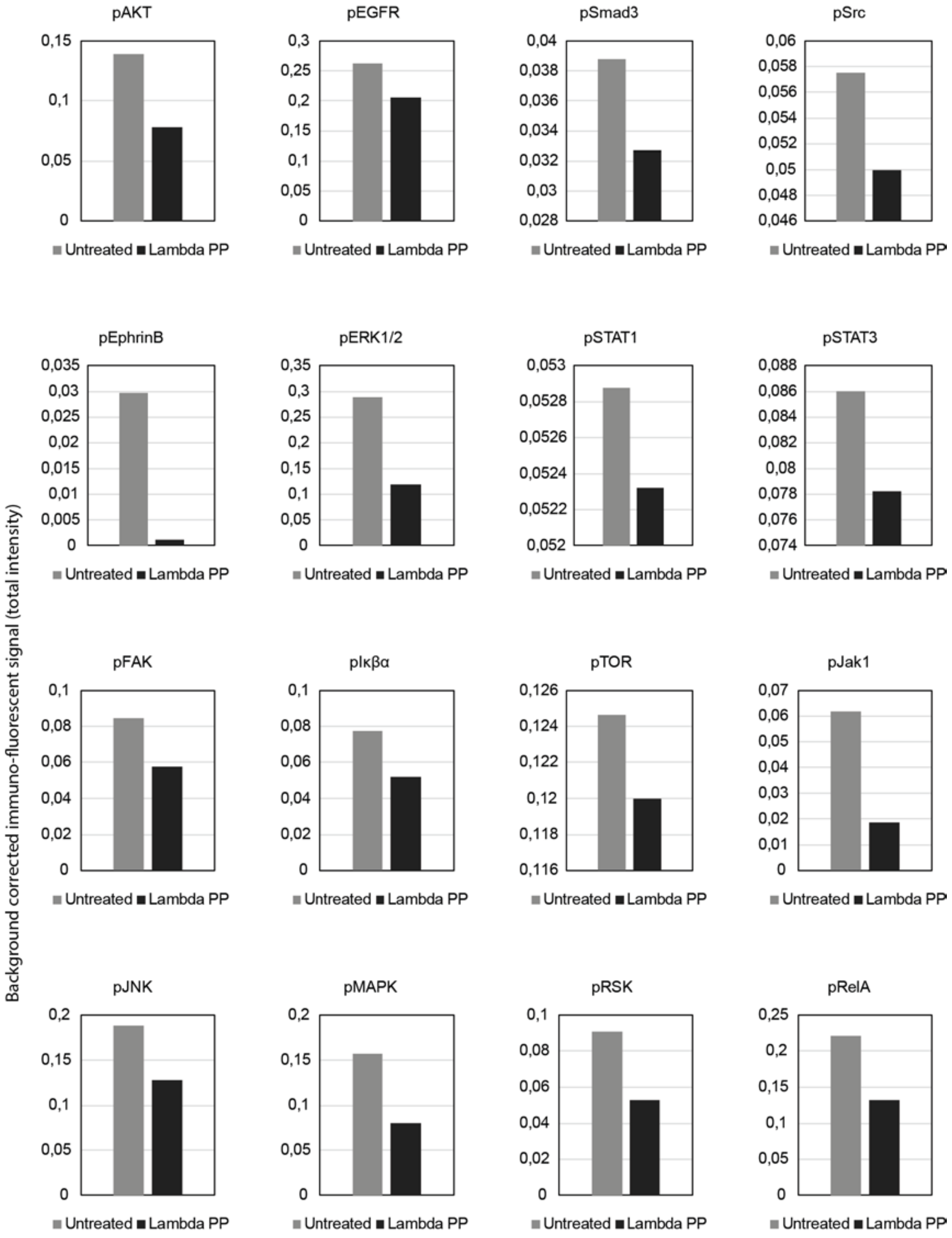

Supplementary Figure 9. Immuno-fluorescence signal after phosphatase treatment of primary keratinocytes. Supplementary Data 1 gives an overview of all antibodies and their corresponding validation experiments. (n=1)

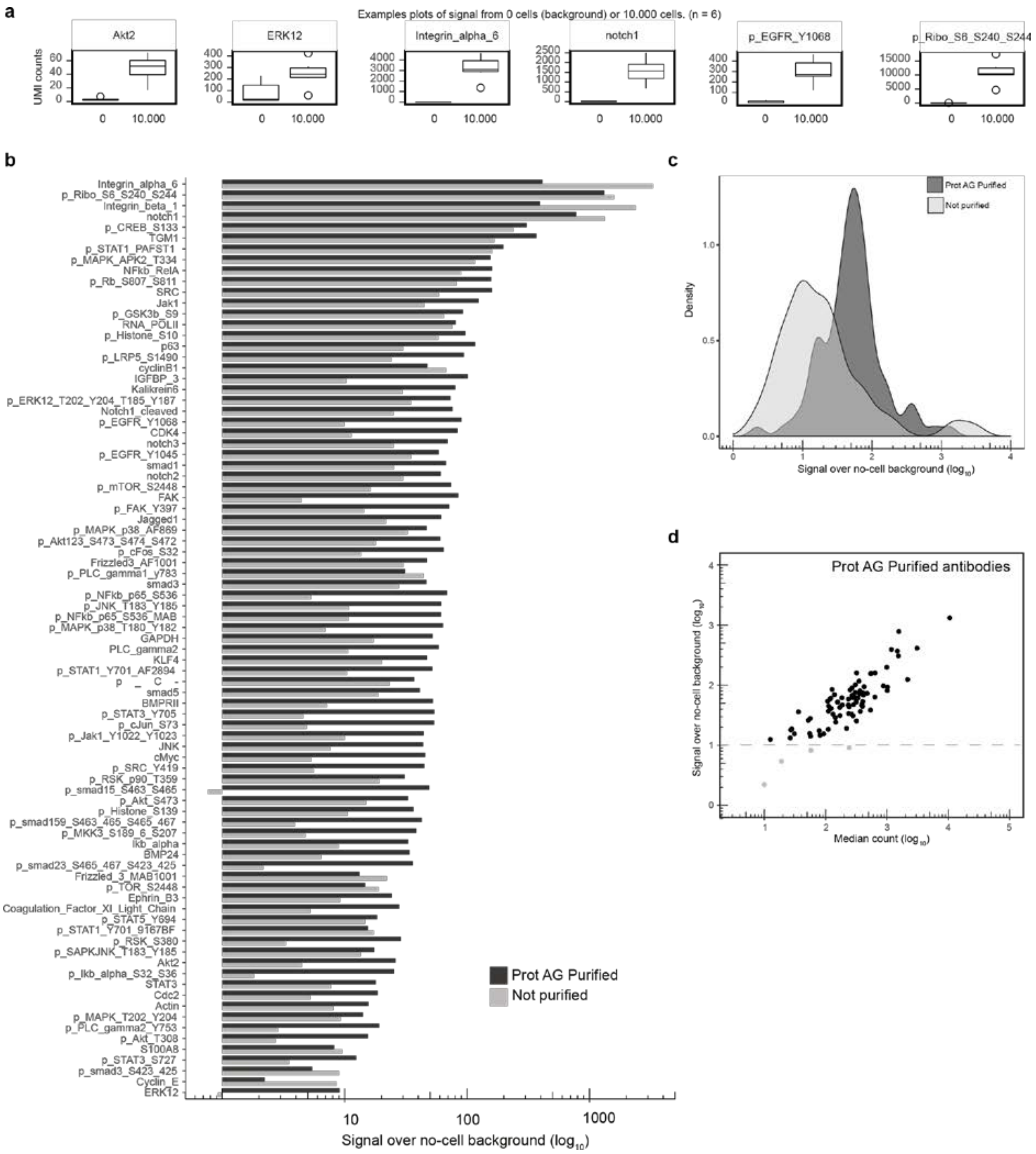

Supplementary Figure 10. Signal-to-noise analysis of two antibody pools (Prot AG purificied and unpurified anitbodies) and dynamic range of antibody signals. **(a)** Boxplots with ID-seq signal from 0 (no-cell background) or 10.000 cells. Selection of 6 different antibodies. Center line indicating median, bounds of boxes showing upper and lower quartile, and whiskers illustrating 1.5*inter-quartile range (n=6). **(b)** Median counts were used to calculate signal-over-background. Light grey bars show signal-over-background from antibodies that were not purified with protein-AG beads. Dark grey bars show signal-over- background from antibodies that were purified with protein-AG beads. **(c)** Density plot of all antibodies` signal over background showing an overall increase for the purified antibody pool. However, several antibodies show decreased signal over background. For PKIS screen, only antibodies that showed increase in signal-over-background were purified over protein- AG beads. **(d)** Scatterplot of median counts against signal over background per antibody. The antibody counts span a range of 3 order of magnitudes. As expected with a relatively stable background measurement, higher counts have higher signal over background.

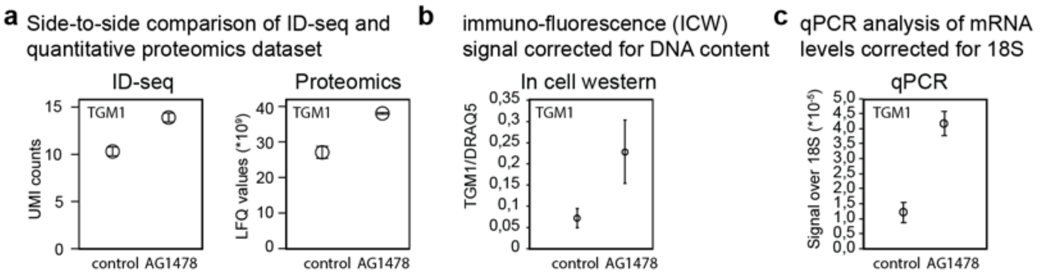

Supplementary Figure 11. TGM1 protein (a and b) and mRNA (c) levels measured using ID-seq (a, n=6) or via quantitative proteomics (a, n=3), via immuno-fluorescence (b, n=4) or RT-qPCR (c, n=3). Similar dynamics can be observed upon AG1478 treatment of keratinocytes. Mean and s.d. are shown in each panel.

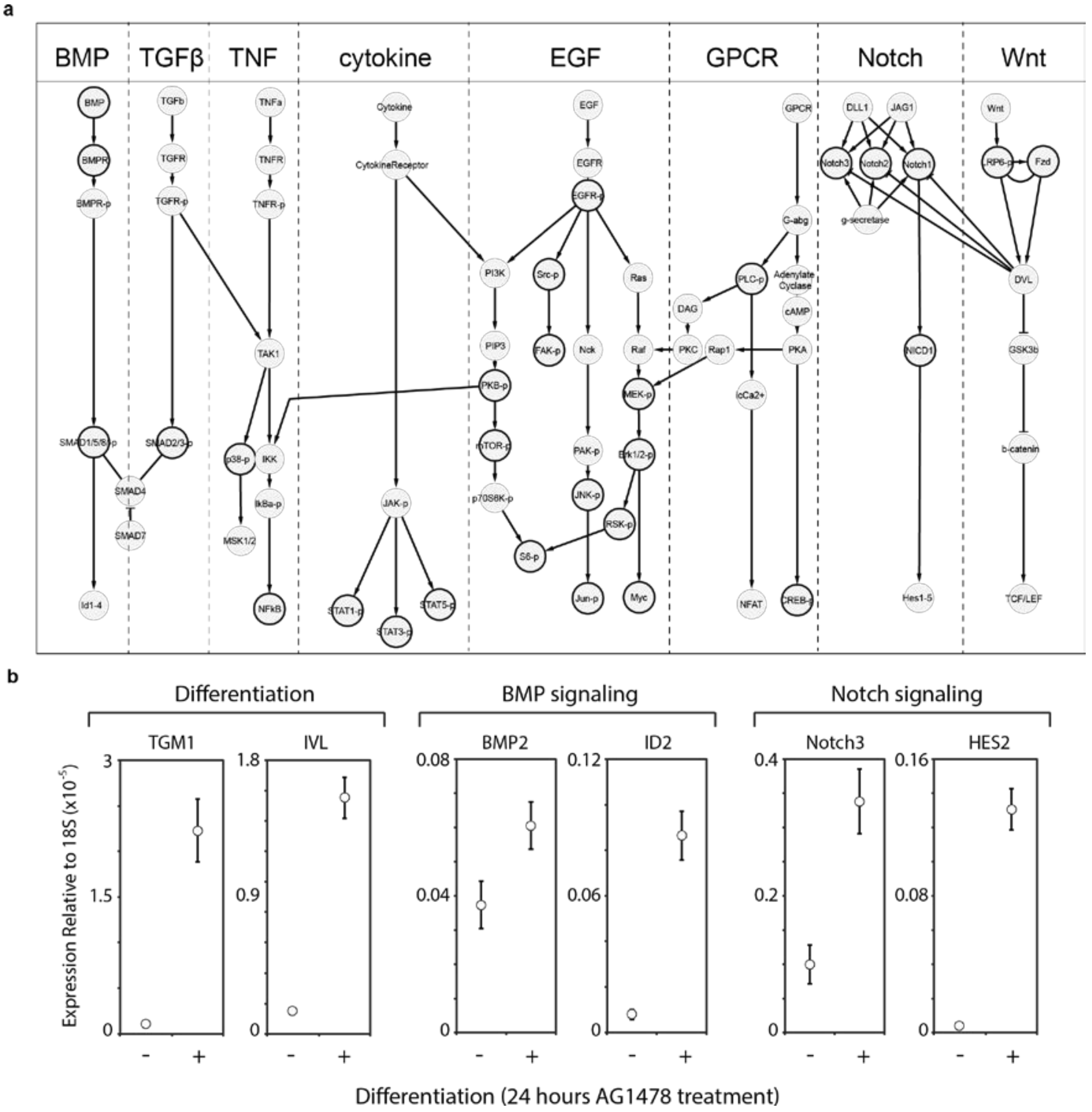

Supplementary Figure 12. Decreased EGF signalling and increased differentiation, BMP and Notch signalling upon AG1478 treatment. (**a**) Cytoscape network of signalling pathways from Figure 1f, including protein names. Only circled proteins were measured in the ID-seq experiment. (**b**) mRNA levels of differentiation markers TGM1 and involucrin (IVL), BMP2 and downstream target ID2, Notch3 and downstream target HES2. (mean and s.d.) Results show that AG1478 treatment (EGFR inhibition) induces differentiation, and increases BMP and notch signaling activity.

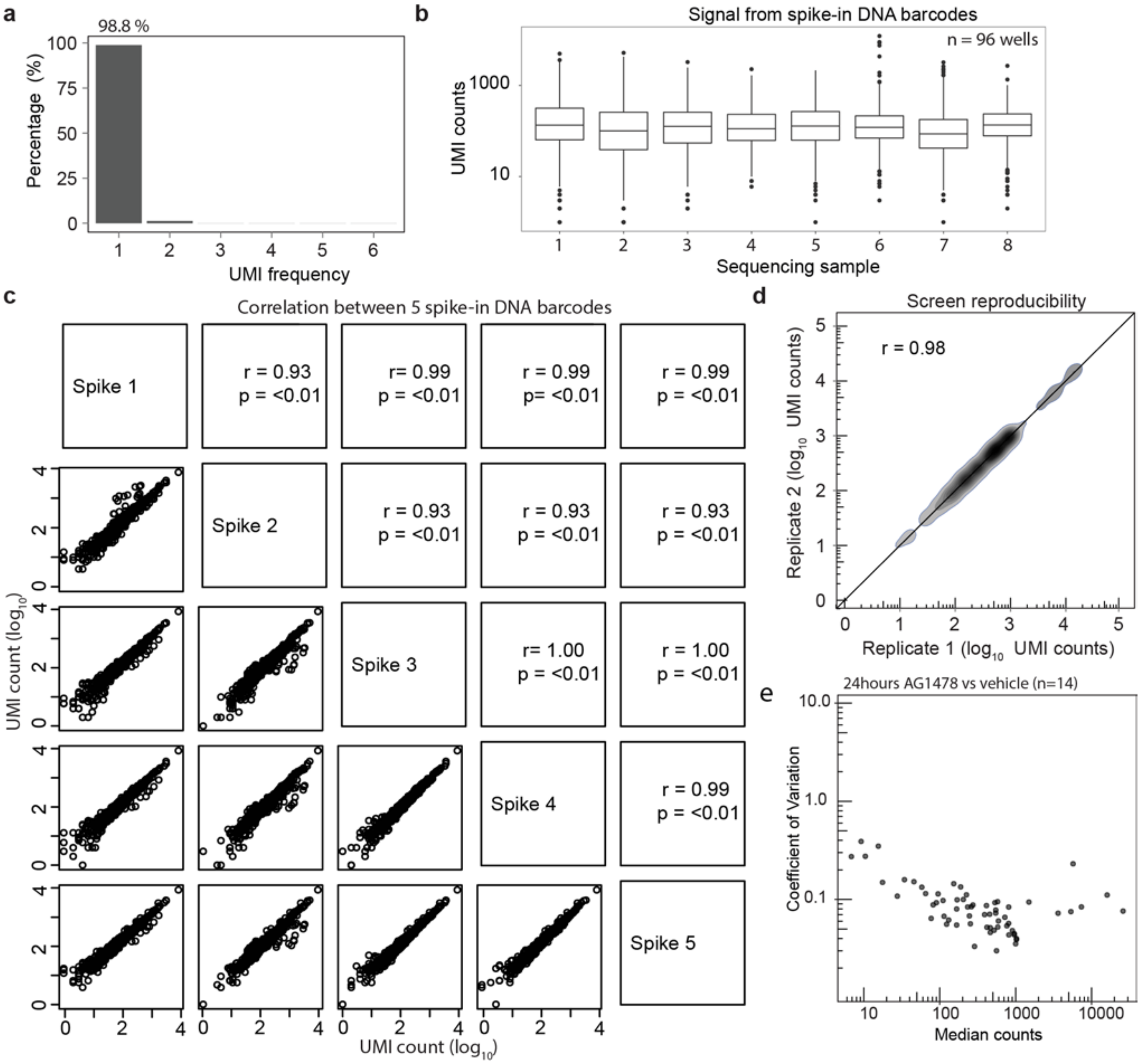

Supplementary Figure 13. Quality controls of the ID-seq PKIS screen. (**a**) Percentage of total reads that are unique (UMI frequency = 1) or are duplicates. (**b**) Spike-in DNA UMI counts from all wells per sequencing sample. The PKIS screen contained 8 sequencing samples, and the boxplot shows that sample-prep for all samples was successful resulting in comparable spike-in DNA counts.

(**c**) Correlation between spike-in DNA barcodes shows reproducible counts for the full screen dataset. (**d**) Counts from two replicate screens correlate well (r = Pearson correlation) illustrating high-quality reproducible ID-seq dataset. (**e**) Median counts versus coefficient of variation (CV) from positive control samples treated with AG1478 (n = 14 replicates) shows over broad range of signal a low CV.

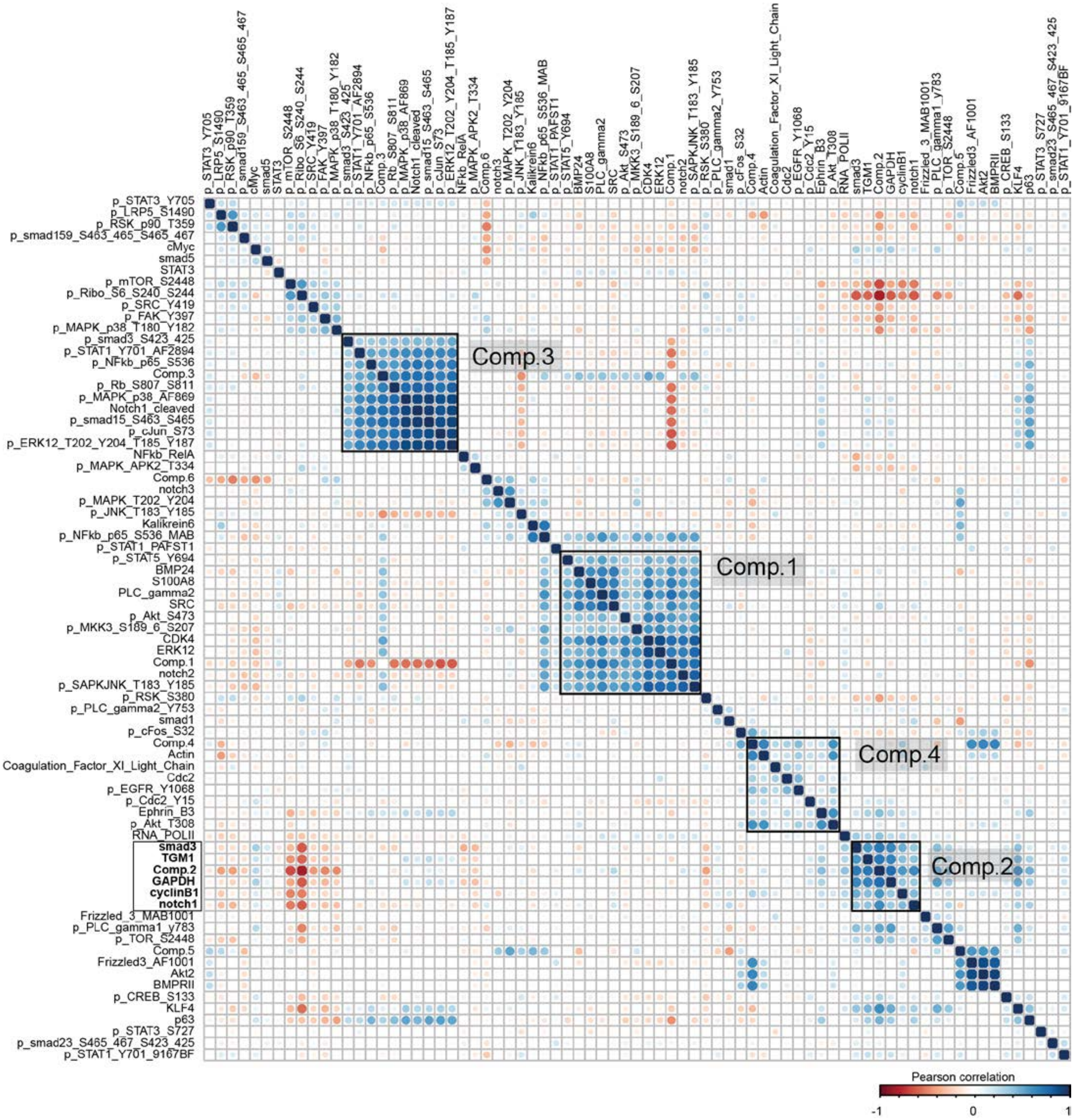

Supplementary Figure 14. Correlations between measured phenotypes and principal components. As described in methods section, PCA analysis was performed using the signed p-values. Here we correlated these signed p-values with the principal components (PCs). PC1 correlates with several signalling events and stress markers. PC2 correlates with differentiation markers TGM1 and Notch1. PC3 correlates with another set of signalling molecules.

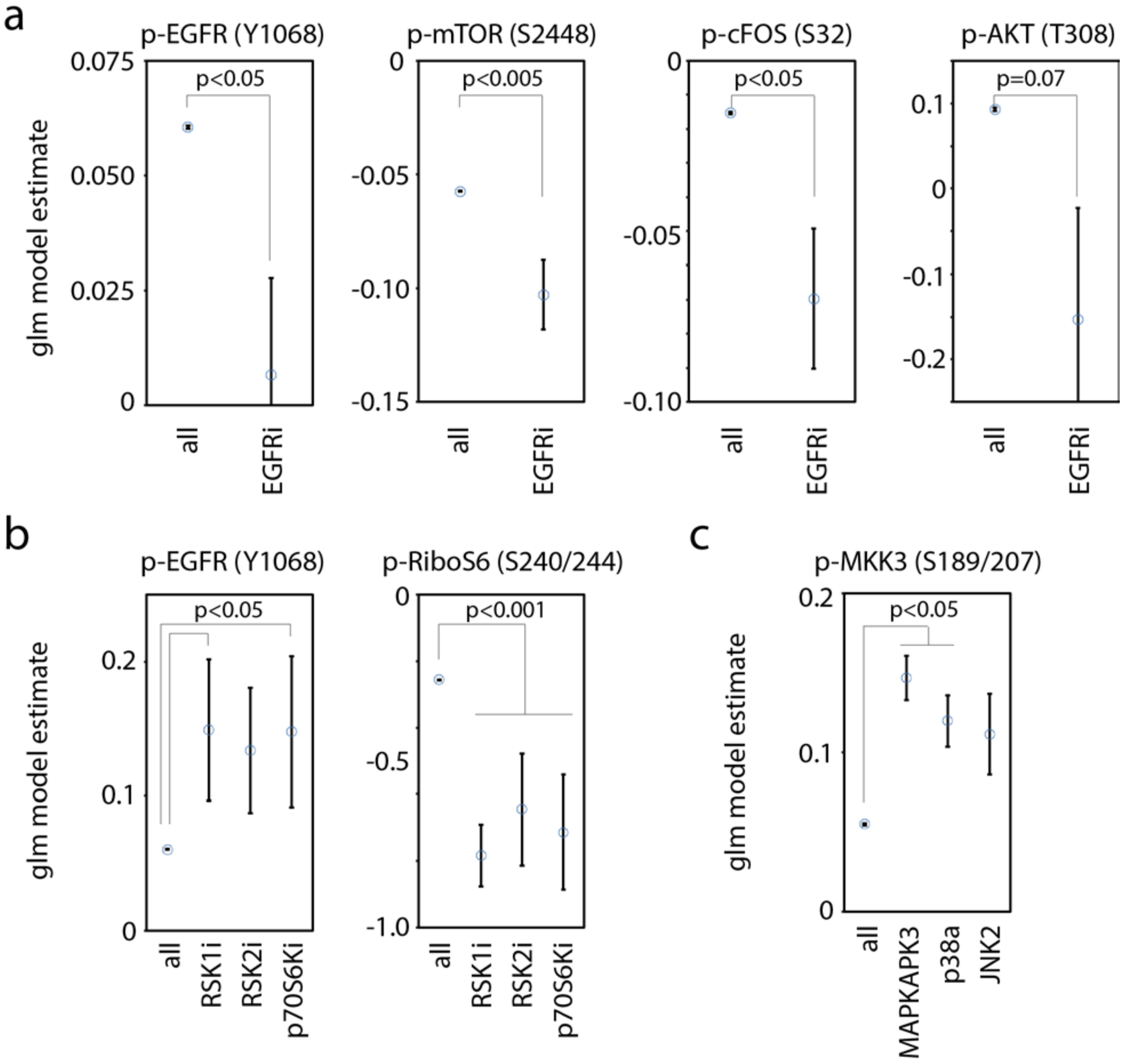

Supplementary Figure 15. Mean ID-seq results of inhibitor sets (x-axis) on indicated phospho-protein levels (panel title). The inhibitor library used for this screen is biochemically tested (by Elkins J. et.al. 2016). Based on this data we assigned (potential) inhibitors to kinases and in this way created 'sets of inhibitors' per kinase. The Supplementary Figure hows for several of these inhibitor sets (EGFRi, RSK1i, RSK2i, p70S6Ki, p38ai, MAPKAPK3i, JNK2i) and the mean ID-seq signal of relevant downstream or upstream (phospho-)proteins. The compounds that can inhibit the EGFR clearly decrease phosphorylation levels of the EGFR, mTOR, cFOS and AKT (a). Interestingly, compound targeting RSK1/2 and/or p70S6K seem to increase phosphorylation of upstream EGFR and decrease S6K phosphorylation (b). Finally, phosphorylation of MKK3 is increased by compounds targeting MAPKAPK3,p38a and/or JNK2 (c). In all, these results confirm the effect of sets of PKIS compounds on relevant compounds. (Mean, s.d., t-test for significance)

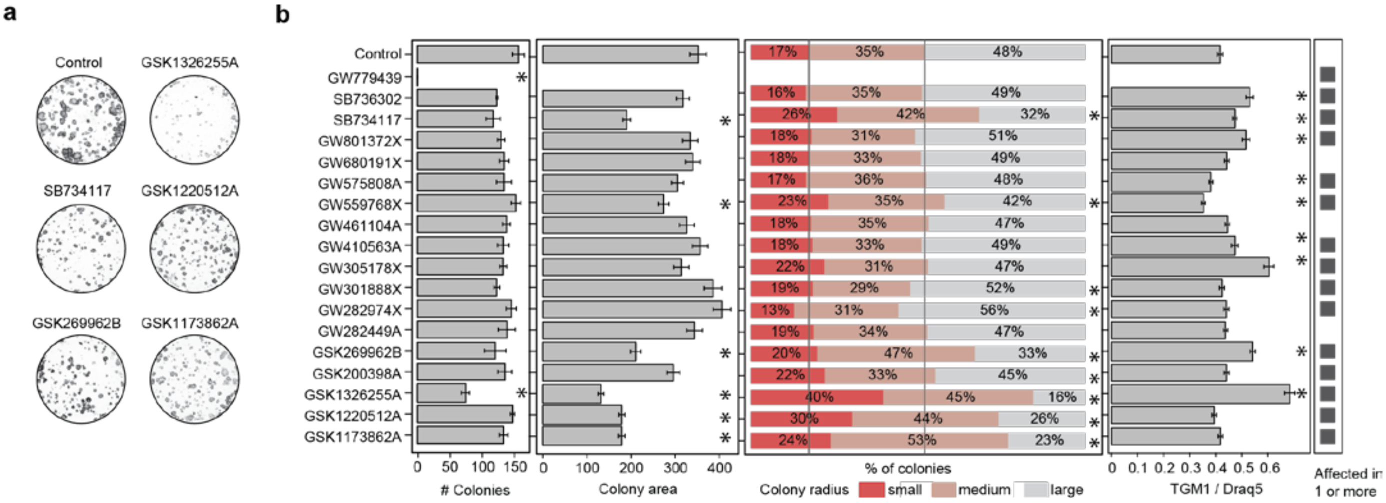

Supplementary Figure 16. Colony formation assay of primary keratinocytes during treatment with selected PKIS compounds (100 nM) shows the effect on colony size and expression of differentiation marker TGM1. **(a)** Representative Draq5 (DNA) staining of colonies. **(b)** Quantification of the number of colonies, colony area, colony area, integrated intensity of TGM1 over Draq5 (n = 3 wells). Dark grey bars show a significant difference to control ($p<0.05$, t-test).

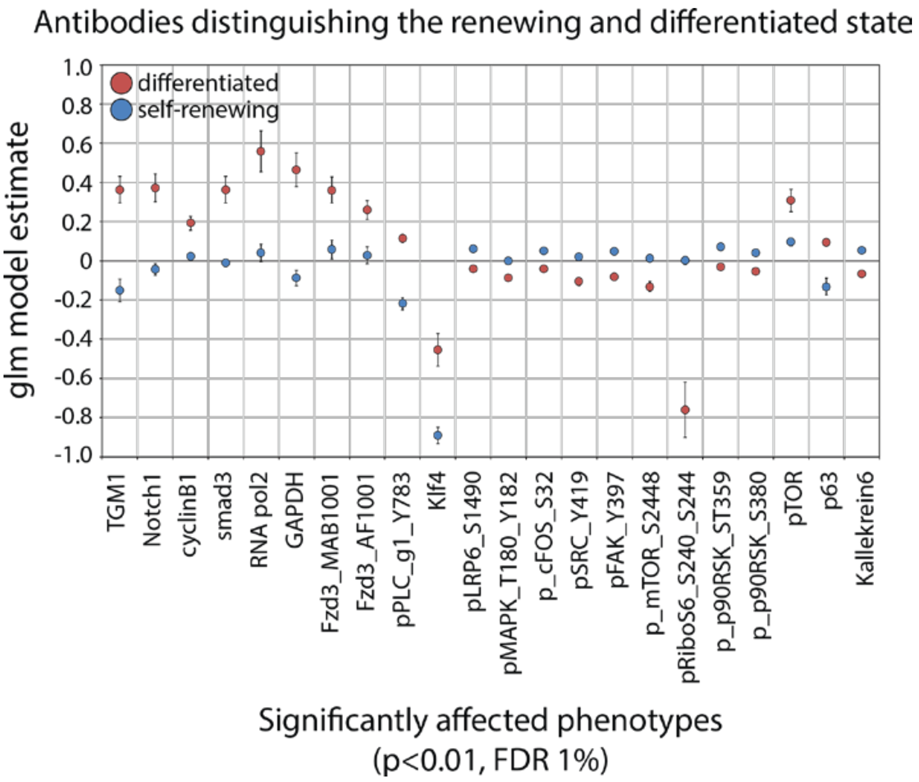

Supplementary Figure 17. Top 10% of probes with high PC2 levels (differentiated) or low PC2 levels (not differentiated/renewal) shows distinct effects on measured phenotypes. The mean and s.d. of the effect on each phenotype is shown: in red the top 10% probes with high PC2 levels ('differentiating') and in blue the bottom 10% probes with low PC2 levels ('renewing').

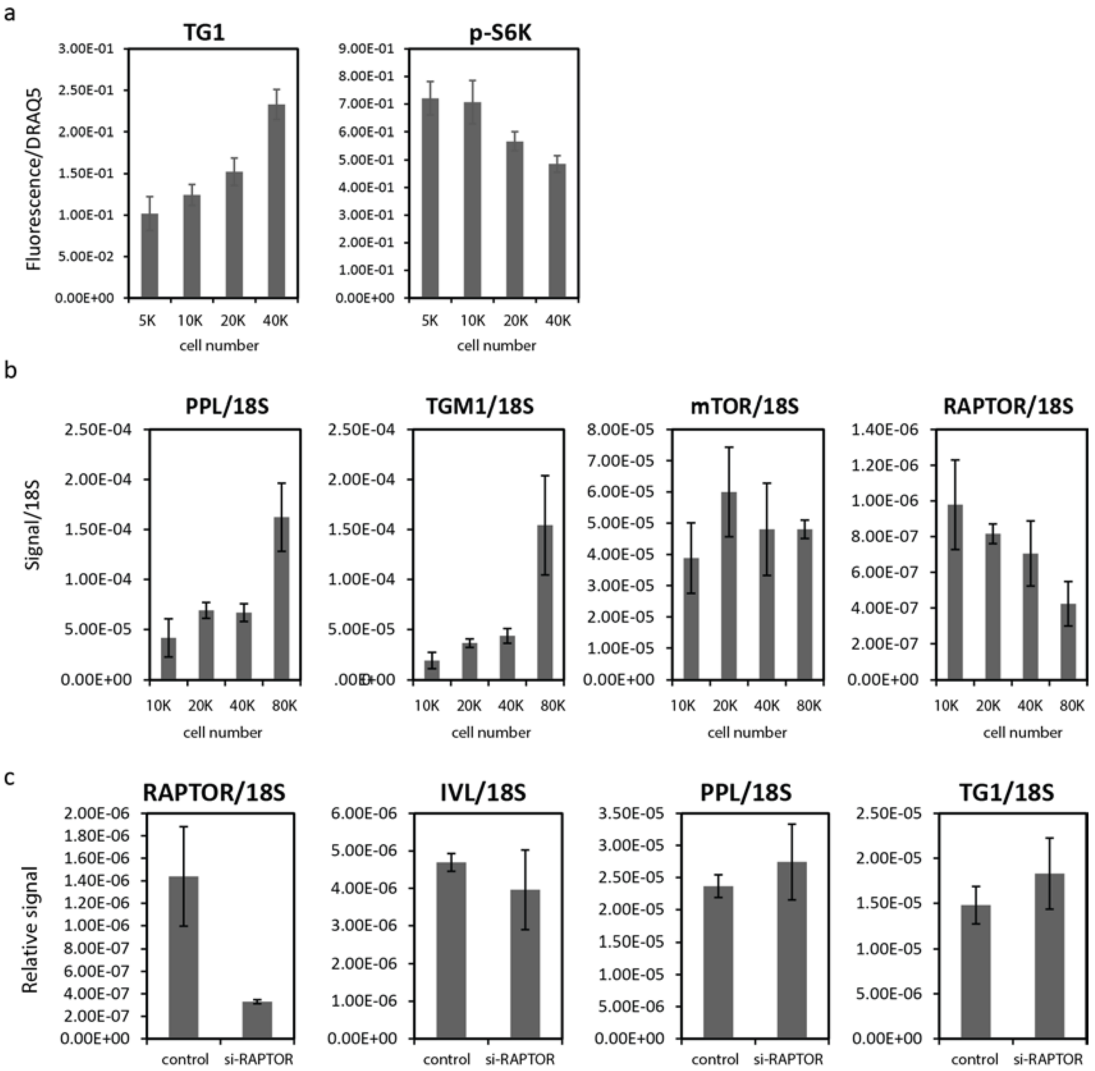

Supplementary Figure 18. Confluency induced differentiating keratinocytes show decreased mTOR signaling activity. (**a**) protein levels measured via in-cell western staining of TG1 and p-S6K from keratinocytes grow at different cell numbers (thousands). Signal intensitiy was corrected for DNA content via DRAQ5 staining. (**b**) mRNA levels of differentiation markers *PPL* and *TGM1*, and signaling components *mTOR* and *RAPTOR*. (**c**) siRNA mediated knockdown of mTOR signaling component RAPTOR does not increase gene expression levels of differentiation markers *IVL*, *PPL* or *TGM1* at 4 days after nucleofection.

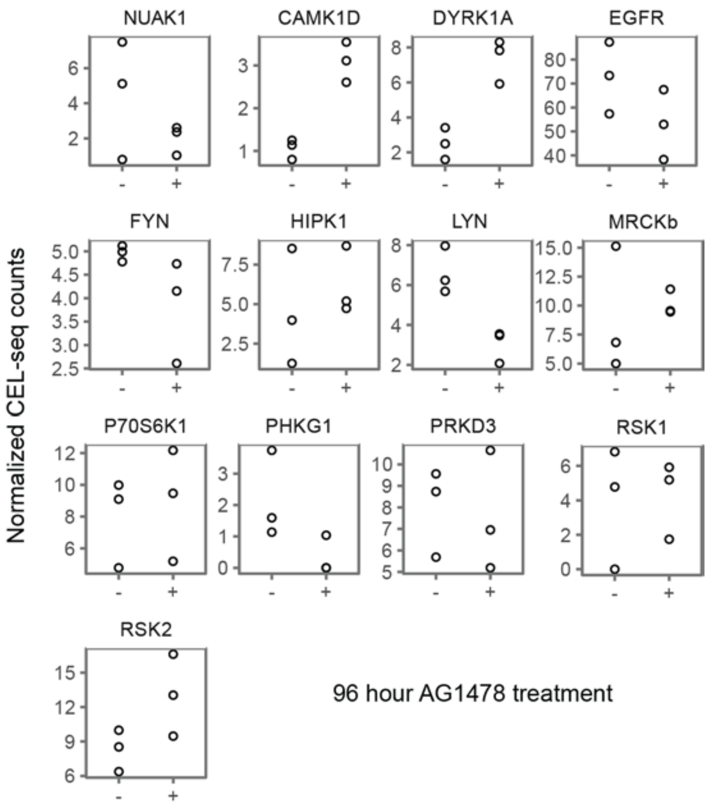

Supplementary Figure 19. Expression levels of enriched kinases in DMSO or 96 hours AG1478 treated primary human keratinocytes. Only kinases that have in all six samples minimum of 1 count in an RNA-seq (CEL-Seq2) dataset were used for further analysis.

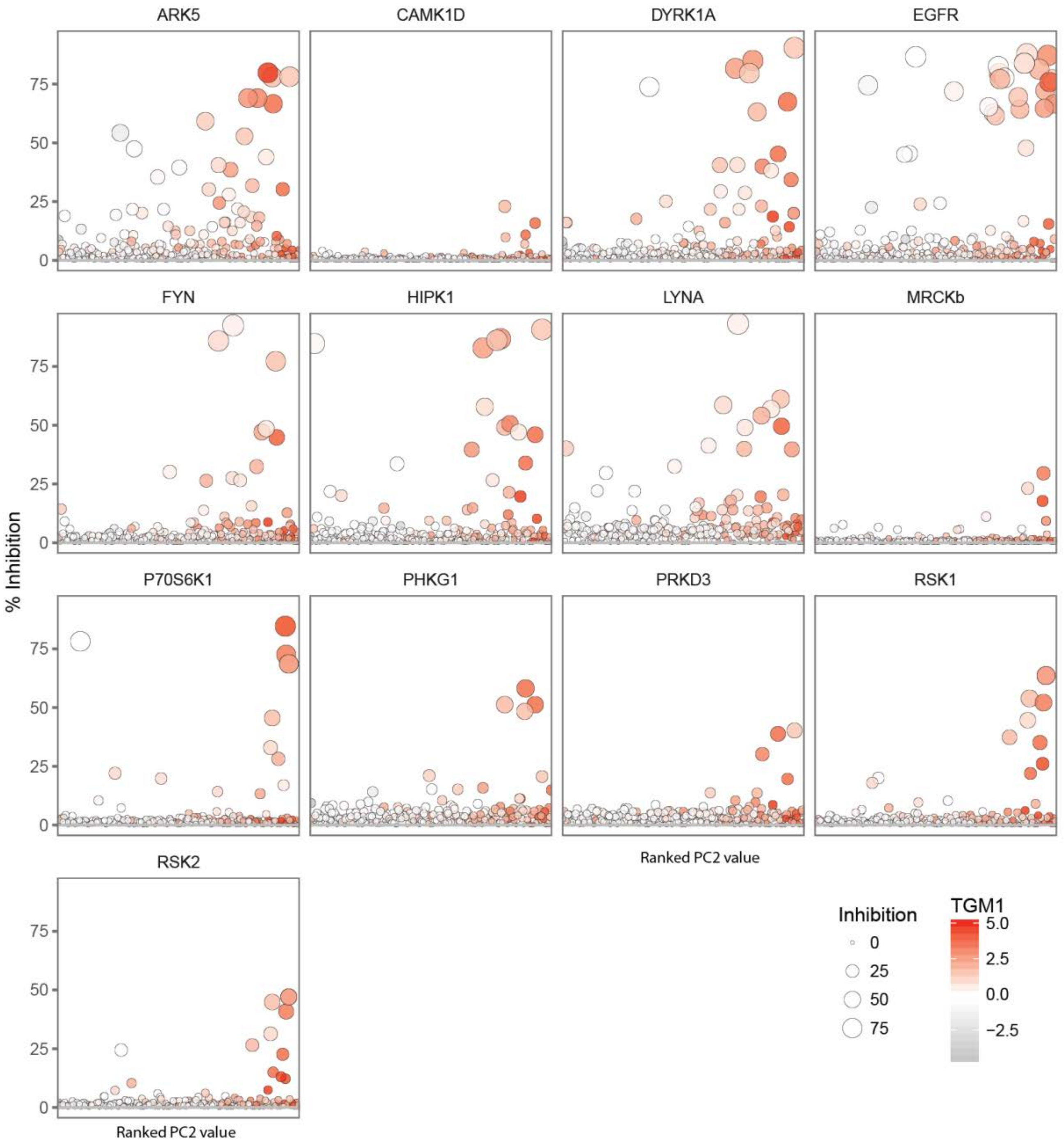

Supplementary Figure 20. Scatterplots with probes ranked on PC2 value (x-axis) and percentage of inhibition for the indicated kinase (y-axis). Colours indicate measured effect (log10 of signed p-value) on differentiation marker TGM1. The plots show that potency of PKIS probes to inhibit the indicated kinases scale with their PC2 score and effect on TGM1.

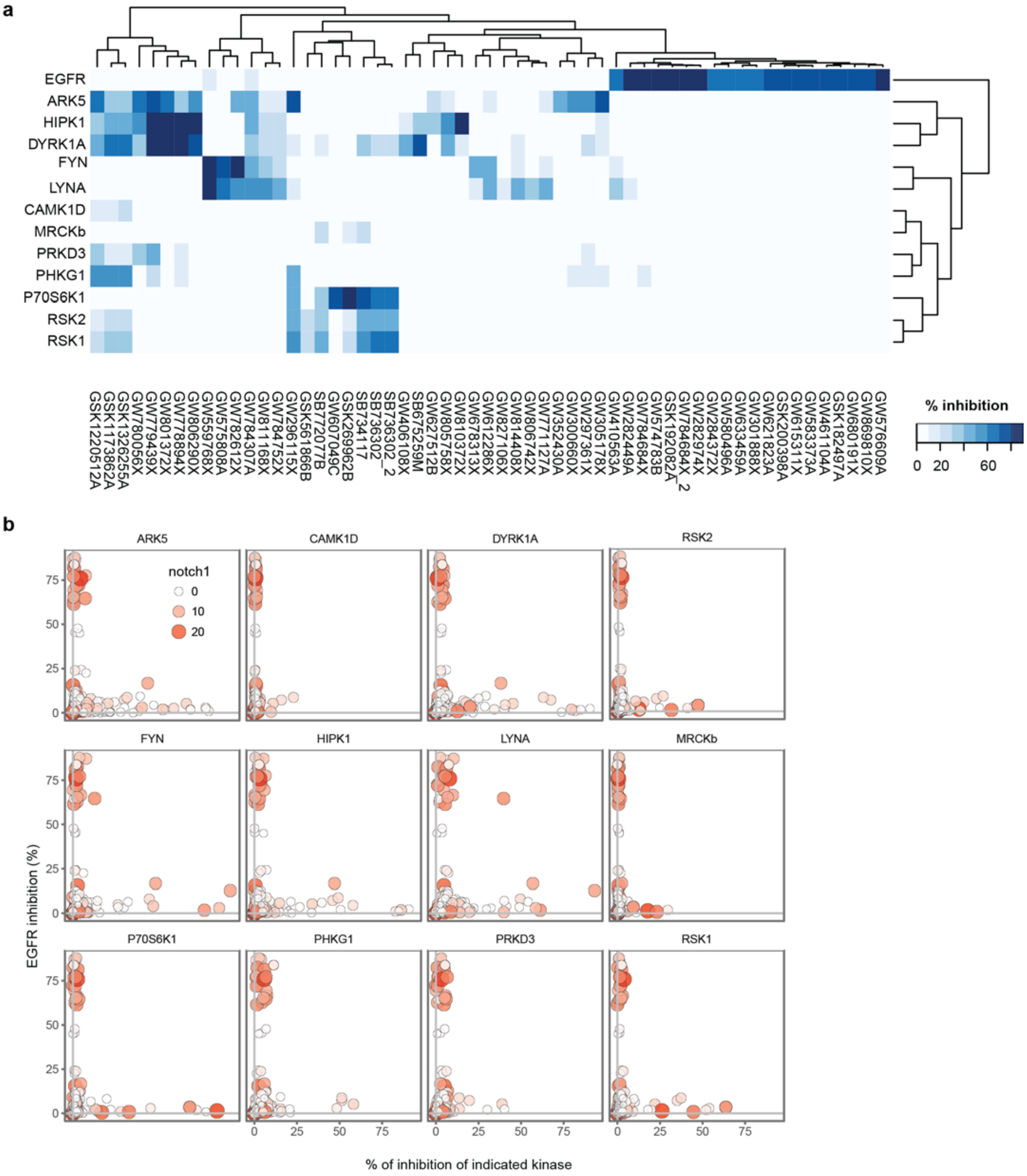

Supplementary Figure 21. Biochemical potency of the probes gives information on which kinases are targeted by the probes in the screen. (**a**) Heat map shows inhibitory effects of enriched probes on the 13 enriched kinases (**b**) Per probe the percentage of inhibition of EGFR is shown (y-axis) and the % of inhibition of the indicated kinases (x-axis). These plots illustrate that the probes targeting the identified enriched kinases do not inhibit the EGFR.

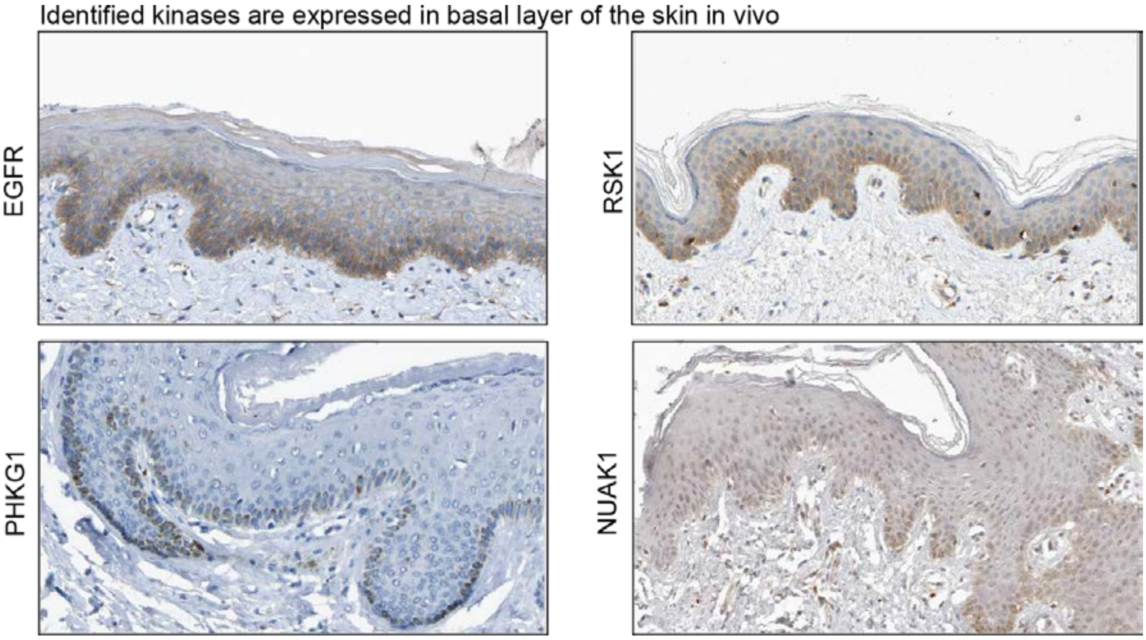

Supplementary Figure 22. Human Protein Atlas[5] immuno-histochemistry staining of EGFR[6], RSK1[7], PHKG1[8] and NUAK1[9] enriched kinases in human skin.

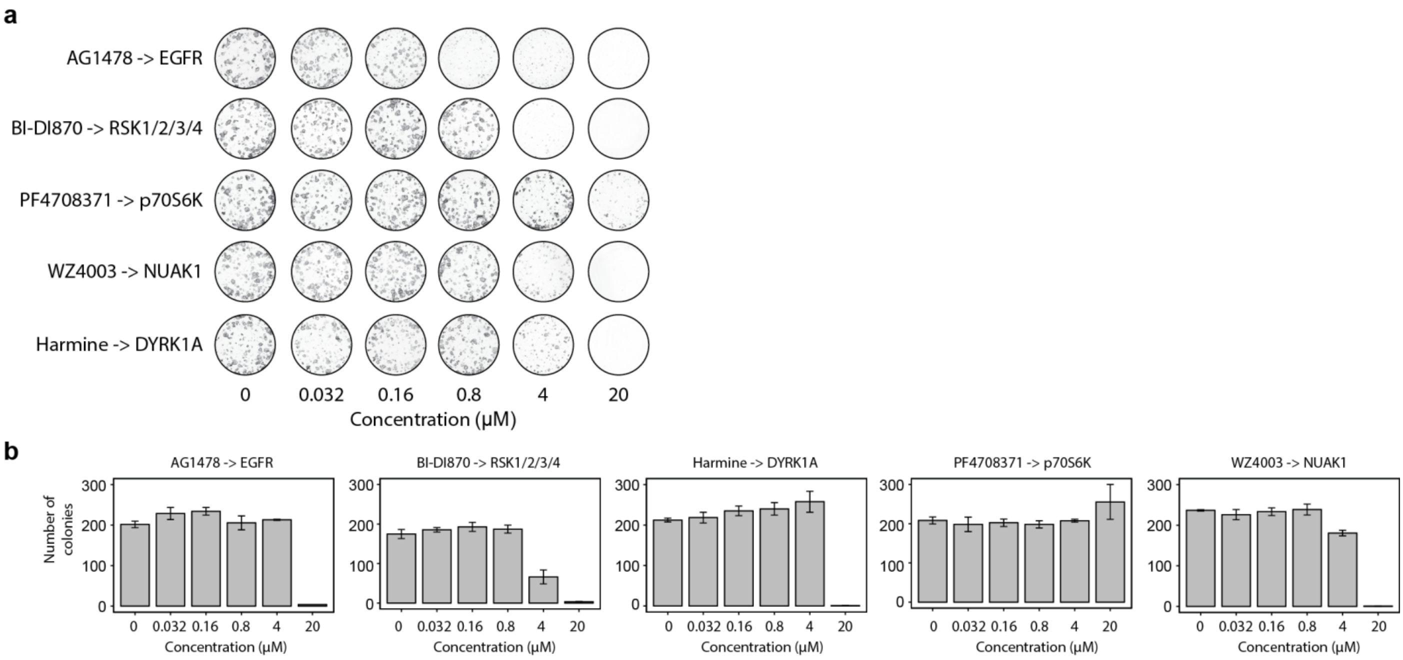

Supplementary Figure 23. Colony forming assay of keratinocytes treated with inhibitors targeting indicated kinases. (a) DRAQ5 DNA staining of colonies per inhibitor -> target, at indicated concentrations. (b) Number of colonies (n=3) per concentration per inhibitor (panel captions), illustrating cell death at 20 μM for 4/5 inhibitors.

Supplementary Tables

Supplementary Table 1. Primers used for qPCR analysis

Gene name	Forward primer	Reverse primer
18S	GTAACCCGTTGAACCCCATT	CCATCCAATCGGTAGTAGCG
TGM1	CCTCACGTTACTGGGAGCAG	TTGCCTCCAATGTCCCCAA
PPL	ATCCGAGAAGCCTCCTCAGT	TTAACGTCCACTCCAGCACC
IVL	TAGGACAGCCAAAGCACCTG	TGAATGTCTTGGACCTGGCC
ITGA6	TTGCGCTCGAGGTTATGGAA	CACAGTCACTCGAACCTGAGT
ITGB1	GCGGACAGTGTGTTTGTAGG	GCAAACACCATTTCCTCCACA
GAPDH	TGACCACCAACTGCTTAG	GATGCAGGGATGATGTTC
P63	TCCATGGATGATCTGGCAAGT	GCCCTTCCAGATCGCATGT
mTOR	ACCCATCCAACCTGATGCTG	ACACTGTCCTTGTGCTCTCG
RAPTOR	TGGGGGACACTGGAAATTCG	TGCTGACAGCTCTGTGTGAG

Supplementary References

1. Andrews, S. FastQC: a quality control tool for high throughput sequence data. Available online at: http://www.bioinformatics.babraham.ac.uk/projects/fastqc. (2010).
2. Love, M. I., Anders, S. & Huber, W. *Differential analysis of count data - the DESeq2 package*. *Genome Biology* **15,** (2014).
3. Anders, S. *et al.* Differential expression analysis for sequence count data. *Genome Biol.* **11,** R106 (2010).
4. Bates, D., Mächler, M., Bolker, B. & Walker, S. Fitting Linear Mixed-Effects Models Using lme4. *J. Stat. Softw.* **67,** 1–48 (2015).
5. Uhlen, M. *et al.* Tissue-based map of the human proteome. *Science (80-.).* **347,** 1260419–1260419 (2015).
6. EGFR image (skin 2, HPA018530) http://www.proteinatlas.org/ENSG00000146648-EGFR/tissue/skin#img available from v16.1 proteinatlas.org.
7. RSK1 image (skin 1 HPA007981) http://www.proteinatlas.org/ENSG00000117676-RPS6KA1/tissue/skin#img available from v16.1 proteinatlas.org.
8. PHKG1 image (skin2 HPA012057) http://www.proteinatlas.org/ENSG00000164776-PHKG1/tissue/skin#img available from v16.1 proteinatlas.org.
9. NUAK 1 image (skin 1, HPA027455) http://www.proteinatlas.org/ENSG00000074590-NUAK1/tissue/skin#img available from v16.1 proteinatlas.org.
