# Supplementary material for: Immuno-detection by sequencing enables large-scale high-dimensional phenotyping in cells
Source: Nat Commun. 2018 Jun 19;9:2384. doi: 10.1038/s41467-018-04761-0 (PMC6008431; doi:10.1038/s41467-018-04761-0)
Supplement: Supplementary file 3 — Description of Additional Supplementary Files [file 41467_2018_4761_MOESM3_ESM.pdf]

Description of Additional Supplementary Files

File Name: Supplementary Data 1
Description: List of all test or used antibodies and additional information including vendor, catalogue numbers, lot numbers, if applicable phospho-sites or clone number, link to datasheets, validation experiments performed and which antibodies were used in each experiment.

File Name: Supplementary Data 2
Description: List of published kinase inhibitor set (PKIS) probes inhibiting a specific kinase.

File Name: Supplementary Data 3
Description: Overview of oligo sequences used to produce DNA-tags, perform sample-prep labelling antibody oligos with DNA-barcodes.
